# *miR-192-2* Regulates the Proliferation and Apoptosis of Ovarian Granulosa Cells by Targeting *IGFBP2* in Zhedong White Geese

**DOI:** 10.3390/ani15050663

**Published:** 2025-02-25

**Authors:** Size Wang, Chuicheng Zeng, Yue Pan, Zhengyu Zang, Yuanliang Zhang, Shan Yue, Xiuhua Zhao, He Huang

**Affiliations:** 1 College of Animal Science and Technology, Northeast Agricultural University, Harbin 150030, China; m15546580001@163.com (S.W.); 18236195872@163.com (C.Z.); 13019655610@163.com (Y.P.); zhengyuzang@126.com (Z.Z.); 2 Animal Husbandry Institute, Heilongjiang Academy of Agricultural Sciences, Harbin 150086, China; zhangyuanliang1218@126.com (Y.Z.); ys924634716@126.com (S.Y.)

**Keywords:** granulosa cells, *miR-192-2*, *IGFBP2*, cell proliferation, apoptosis, goose

## Abstract

**Simple Summary:**

The proliferation and apoptosis of ovarian granulosa cells (GC) is critical for egg-laying performance. In geese, follicular atrophy is accompanied by follicular GC apoptosis during brooding stages. MicroRNAs are involved in follicular development, atrophy, ovulation, and degeneration. Our previous high-throughput sequencing study of goose ovaries from laying and brooding geese revealed that *miR-192-2* may be involved in follicle cell proliferation and apoptosis. The aim of this study was to explore the effects of *miR-192-2* and its target gene *IGFBP2* on the proliferation and apoptosis of follicular granulosa cells in Zhedong white geese. The results showed that after *miR-192-2* overexpression, the expressions of *PCNA*, *CDK2*, *CCND1*, *CCND2*, and *Bcl-2* were significantly decreased, and the expressions of *Caspase3*, *Caspase8*, and *Caspase9* were significantly increased. While the expressions of *PCNA*, *CDK2*, *CCND1*, *CCND2*, and *Bcl-2* were significantly decreased after the downregulation of *IGFBP2* expression, the expressions of *Caspase3*, *Caspase8*, *and Caspase9* were significantly increased. In conclusion, *miR-192-2* inhibited proliferation and promoted apoptosis of follicular GC by targeting *IGFBP2* in Zhedong White geese. Therefore, it is possible to explore inhibiting the expression of *miR-192-2* in production to alter the brooding behavior of Zhedong White Geese, thereby improving egg production and economic returns for producers.

**Abstract:**

The proliferation and apoptosis of ovarian granulosa cells (GC) is critical for follicular development and ovulation, especially for egg-laying performance in female birds. In geese, follicular atrophy is accompanied by follicular GC apoptosis during brooding stages. MicroRNAs are involved in follicular development, atrophy, ovulation, and degeneration. Our previous high-throughput sequencing study of ovaries from laying and brooding geese revealed that *miR-192-2* may be involved in follicle growth and development, as well as follicle cell proliferation and apoptosis in geese. To further investigate the effect of *miR-192-2* on GC in geese, we screened the target gene of *miR-192-2* (*IGFBP2*) and constructed a *miR-192-2* overexpression and interference vector, synthesized a small interfering RNA for *IGFBP2*. The results showed that after *miR-192-2* overexpression, the mRNA and protein expression of proliferation-related genes (*PCNA*, *CDK2*, *CCND1*, and *CCND2*) were significantly decreased, the mRNA and protein expression of apoptosis-related genes (*Caspase3*, *Caspase8*, and *Caspase9*) were significantly increased, and the mRNA and protein expression of anti-apoptosis gene *Bcl-2* were significantly decreased. While the mRNA and protein expressions of *PCNA*, *CDK2*, *CCND1*, and *CCND2* were significantly decreased after the downregulation of *IGFBP2* expression, the mRNA and protein expressions of *Caspase3*, *Caspase8*, and *Caspase9* were significantly increased, and the mRNA and protein expression of *Bcl-2* was significantly decreased. In conclusion, *miR-192-2* inhibited proliferation and promoted apoptosis of follicular GC by targeting *IGFBP2* in Zhedong White geese. Apoptosis of GC leads to follicular atresia, which in turn leads to brooding behavior in female geese. Therefore, it is possible to explore inhibiting the expression of *miR-192-2* in production to alter the brooding behavior of Zhedong White Geese, thereby improving egg production and economic returns for producers.

## 1. Introduction

Follicular development is a key physiological process in the reproductive system of female animals [1]. During the reproductive cycle of poultry, there are a large number of primordial follicles in the ovary, but only a few follicles undergo selective development to eventually mature and ovulate, while the other unselected follicles gradually shrink and degenerate in a process known as follicular atrophy [2]. Granulosa cells (GC) are the essential cells that make up the follicle [3], and GC differentiation, proliferation, and apoptosis are closely linked to primordial follicle activation, follicular growth, and follicular expulsion and atrophy [4,5]. In developing follicles, the number of apoptotic GC accounts for 10% of the total number of GC before follicular atrophy [6]. The apoptosis of GC is regulated primarily by their autocrine paracrine secretion pattern, but also by combinations of several cytokines, hormones, and growth factors [7]. The apoptosis of GC is also the main cause of ovarian atrophy, which leads to brooding behavior in the female goose [8].

miRNAs are endogenous single-stranded non-coding RNAs of approximately 20–24 nt in length [9]. miRNAs contain structures complementary to target genes and enable the transcriptional or post-transcriptional regulation of target genes, thereby regulating gene expression and various biological processes [10]. miRNAs play extremely important roles in various stages of follicular development [11], such as the regulation of follicular growth, follicular atresia, dominant follicle formation, and ovulation, as well as the regulation of the proliferation and apoptosis of GC [12]. *miR-192-2* is a member of the *miR-192* family, which has been implicated in many studies as a potential prognostic and diagnostic biomarker and even as a therapeutic target for a variety of cancers [13]. According to a recent report, *miR-192* (enriched in hepatic stellate cells) associated with hepatocellular carcinoma can inhibit hepatic stellate cell activation by directly targeting Rictor in the *AKT/mTORC2* pathway [14]. There was also evidence that *miR-192* can regulate the proliferation or apoptosis of porcine uterine epithelial cells by directly targeting and inhibiting the expression of *CSK* and *YY1* genes [15]. Another study showed that the overexpression of *miR-192* inhibited the proliferation of hepatocellular carcinoma cells and promoted apoptosis [16]. According to two other studies, *miR-192* was identified as a prognostic marker for ovarian tumor samples [17]. Moreover, the damage of granulosa cells induced by oxidative stress can be reduced by the downregulation of *miR-192* in pigs [18]. These studies suggested that *miR-192* may play a role in ovarian development in animals, as well as influencing cell proliferation and apoptosis. However, the potential regulatory mechanisms of *miR-192-2* in GC proliferation and apoptosis are unknown.

Insulin-like growth factor-binding protein 2 (*IGFBP2*) is a member of the family of insulin-like growth factor-binding proteins, which are highly conserved in many complex life processes [19,20,21]. Previous studies had shown that *IGFBP2* was associated with ovarian cancer and overexpressed in malignant ovarian tissue as well as in the serum and cystic fluid of ovarian cancer patients, suggesting that *IGFBP2* plays an important role in ovarian cancer biology [22]. In ovarian cancer cells, *COL11A1* had been shown to regulate the *TGF-β3* activation of cancer-associated fibroblasts through the *NF-κB/IGFBP2* axis [23]. Although *IGFBP2* is involved in many cellular processes, its role in the mechanism of ovarian GC requires further investigation. As mentioned earlier, *miR-192* and *IGFBP2* both have specific functions in animal ovaries, but is there a connection between them? If there is a connection, how is it regulated between them? These are the topics that this study aims to investigate.

In geese, the specific function of *miR-192-2* in GC is unknown. Therefore, this study is to investigate the potential function of *miR-192-2* in goose GC and its undiscovered regulatory mechanisms. It may provide new insights from a microRNA perspective on mitigating brooding behavior and improving egg production in geese.

## 2. Materials and Methods

### 2.1. Experimental Animals

The experimental animals were provided by Zhejiang Xiangshan Wenjie White Goose Co., Ltd. (Xiangshan, Zhejiang, China) The geese were 18-month-old female geese with similar body weight. They were treated with the same feeding conditions, free water feeding, natural light, and ventilation. Twenty female geese were selected from each of the laying and brooding periods. The present study was conducted under the approval of the Institutional Animal Care and Use Committee of Northeast Agricultural University (approval number: SRM-06, approval date: 1 January 2018) and in accordance with the laboratory animal guidelines for ethical review of animal welfare (GB/T 35 892-2018) [24].

### 2.2. GC Isolation and Culture

Passage cells were from female goose ovarian primary granulosa cells. After the experimental animals were killed by cervical dislocation, the surface envelope of the ovary, the surrounding connective tissue, and the adipose tissue mass were removed with forceps, and GC were collected according to Gilbert’s method [25]. GC were obtained by digestion with 0.1% collagenase (Sigma, Aldrich, St. Louis, MO, USA). GC were incubated in Dulbecco’s modified Eagle’s medium/F12 (DMEM/F12, Hyclone, Logan, UT, USA) containing 10% fetal bovine serum (FBS) (Gibco, Carlsbad, CA, USA) and 1% streptomycin and penicillin mixture (Gibco, Carlsbad, CA, USA) at 37 °C and 5 °C, respectively. The total number of cells cultured in the medium dish was 3 × 10^5^. GC were cultured in 12-well plates until they were 70–80% confluent (3 × 10^4^ cells/well) with 3 technical replicates per group.

### 2.3. Transient Transfection, RNA Isolation, and RT-qPCR

*miR-192-2* mimic, *miR-192-2* inhibitor, miRNA mimic negative control (mimic NC), and miRNA inhibitor negative control (inhibitor NC) were purchased from Qingdao Biotechnology Co. (Qingdao, China) Goose *IGFBP2* siRNA and siRNA negative control were synthesized by GenePharma (GenePharma, Shanghai, China). Wild type (WT) and mutant (MT) *IGFBP2* 3′UTR with *miR-192-2* binding sites were synthesized and inserted into the 3′ region of the firefly luciferase gene, a pmirGLO dual luciferase reporter vector containing the Renilla luciferase gene cassette, for a dual luciferase reporter gene assay (Promega, Madison, WI, USA). These small oligonucleotides were transfected into cells at different concentrations using Lipofectamine 2000 reagent, and GC and 293T were inoculated into DMEM/F12 (DMEM/F12, Hyclone, Logan, UT, USA) medium in 12-well and 96-well plates, respectively, with 10% bovine serum (Gibco, Carlsbad, CA, USA) and 1% antibiotics (Gibco, Carlsbad, CA, USA), and 1% antibiotics (1% penicillin and 1% streptomycin mixture, Gibco, Carlsbad, CA, USA). When the cells reached 70–80% concentration, different concentrations of miRNA were transfected into the cells. We performed a gradient screening of *miR-192-2* mimics and *miR-192-2* inhibitors at concentrations ranging from (20 nM, 30 nM, 50 nM, 100 nM, and 200 nM), respectively. We tested and screened the most suitable concentrations for transfection experiments. The optimal expressions of *miR-192-2* mimic and *miR-192-2* inhibitor were reached at 50 nM and 100 nM, respectively. All experiments were replicated three times. After transfected cells were cultured, total RNA was isolated using TRIzol reagent (Takara, Shiga, Japan), and reverse transcription was performed using TransScriptVR One-Step gDNA Removal and cDNA Synthesis SuperMix (TransGen, Beijing, China). The RT-qPCR reaction system was 10 μL, including 1 μL cDNA, 0.5 μL forward and reverse primers, 5 μL TB Green TM Premix (Takara, Shiga, Japan), and 3 μL DNase/rase-free deionized water (Tiangen, Beijing, China). The β-actin and U6 genes were selected as internal reference genes. Primer information is shown in Table 1 and Table 2. The relative expression of the RT-qPCR data was analyzed using the 2^−∆∆Ct^ method.

### 2.4. Western Blot Analysis

Proteins were extracted from goose ovarian GC 48 h after transfection. The protein concentration was determined using a BCA protein concentration assay kit (SW101, Seven Countries, China), and after the concentration was standardized, 5 × SDS supersampling buffer (Beyotime, Shanghai, China) was added, boiled, and stored at −20 °C. Water bath for 5 min. Proteins were separated by 10% sodium dodecyl sulfate PAGE gel electrophoresis and transferred to a PVDF membrane (Beyotime, Shanghai, China). Skimmed milk 5% (diluted with TBST) was added for 2 h and then incubated with primary antibody at 4 °C overnight. The strips were washed three times with TBST for 8 min each, and the anti-rabbit secondary antibody was incubated for 2 h. The strips were washed three times with TBST for 8 min each. The strips were detected using a molecular imaging system (SmartchemiTM Image Analysis System, Beijing Shengchuang Science and Technology Co., Ltd., Beijing, China). The strips were analyzed using Image J software (v1.8.0 NIH, Bethesda, MD, USA), and the peak plots were used to get the area value for each peak with three technical replicates per group.

### 2.5. Flow Cytometry

Cell suspensions were digested with 0.25% trypsin-EDTA and stained with an Annexin V/PI Apoptosis Detection Kit (Nantong Beiten Biotechnology, Nantong, Jiangsu, China) according to the instructions to detect apoptotic levels. Apoptotic cells were quantified using a BD Accuri C6 flow cytometer (Thermo Scientific, Waltham, MA, USA), and data were analyzed using FlowJo software (v10.8.1), and the sum of early and late apoptotic subpopulations was then calculated as the apoptosis rate.

### 2.6. Dual Luciferase Assay

The predicted target genes of goose *miR-192-2* regulating the proliferation and apoptosis of GC were analyzed by miRDB (http://mirdb.org/, accessed on 1 October 2023) and TargetScan (https://www.targetscan.org/vert_80, accessed on 1 October 2023) and their crosstalk with DEGs. Human Embryonic Kidney 293T cells were maintained in DMEM/F12 medium (Hyclone, Logan, UT, USA) supplemented with 10% neonatal bovine serum (Gibco, Carlsbad, CA, USA), 100 μg/mL streptomycin, and 100 IU/mL penicillin. Cells were incubated at 37 °C saturated humidity, 5% CO_2_, and the medium was changed every 24 h. Then, 293T cells in 48-well plates were co-transfected with plasmids (wild-type or mutant pmiRGLO-3′UTR-*IGFBP2*) (2 µg) and *miR-192-2* mimic or mimic-nc (2 µL), and luciferase activity was detected using a dual luciferase reporter assay system (Beyotime, Shanghai, China). Relative luciferase activity was determined by normalizing the luciferase activity of fireflies to that of kidney cells.

### 2.7. EdU Assay

A Cell-LightTM EdU Apollo VitroKit (RiboBio, Guangzhou, China) was used for the GC proliferation assay. The EdU incorporation rate was calculated as the ratio of the number of EdU incorporated cells to the number of Hoechst 33,342 stained cells according to the following formula. A minimum of 500 cells were counted in each group.

$$\text{EdU}\;\text{incorporation}\;\text{rate}=\frac{\text{EdU}\;\text{incorporated}\;\text{cells}}{\text{Hoechst}\;33,342\;\text{stained}\;\text{cells}}$$

### 2.8. CCK-8 Assay

Cell activity in GC was detected using a Cell Counting Kit-8 assay (CCK-8 kit, Houston, TX, USA). The collected primary GC of goose follicles were cultured in 96-well plates and 100 µL of cell suspension was added to each well. The absorbance of the cells at 450 nm was measured using an enzyme marker at the end of transfection and the data were recorded.

### 2.9. Statistical Analysis

All results were expressed as mean ± SEM. Western blot results were analyzed using Image J software. Experimental data were analyzed using SPSS 20.0 software, using ANOVA and Dunnett’s test or Tukey’s multiple comparisons, and plotted using GraphPad Prism 8 software.

## 3. Result

### 3.1. miR-192-2 Inhibits Proliferation of Ovarian Follicular Cells

**Figure 1:**
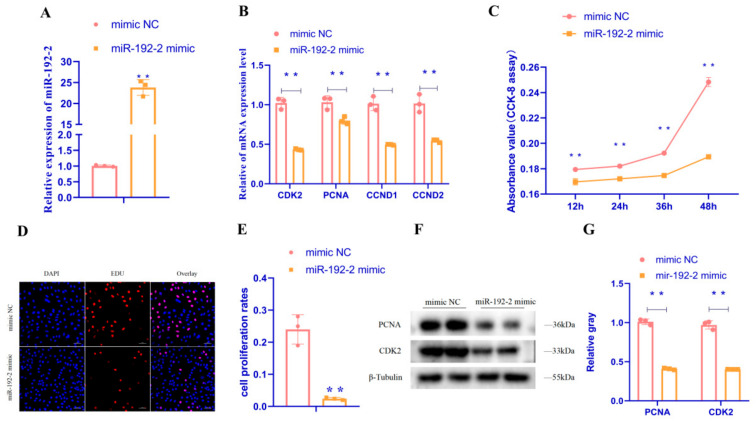
*miR-192-2* mimic regulates the proliferation of goose follicle granulosa cells. (**A**) After transfection with *miR-192-2* mimics or negative controls (mimic NC), *miR-192-2* expression in granulosa cells was monitored by qRT-PCR. (**B**) mRNA expression of *CDK2*, *PCNA*, *CCND1*, and *CCND2* in granulosa cells transfected with a *miR-192-2* mimic or mimic negative controls. (**C**) Granulosa cells were transfected with *miR-192-2* mimic or mimic negative controls. Cell growth curves were determined using CCK-8. (**D**,**E**) The proportion of cells in a proliferative state was determined after transfection of *miR-192-2* mimic or mimic negative controls into granulosa cells using EdU (Scale bar = 50 μm). (**F**,**G**) Protein expression of *CDK2* and *PCNA* in granulosa cells was detected by WB after transfection of *miR-192-2* mimic or mimic negative controls into granulosa cells. Data are expressed as mean ± SEM (*n* = 3). ** *p* < 0.01.

**Figure 2:**
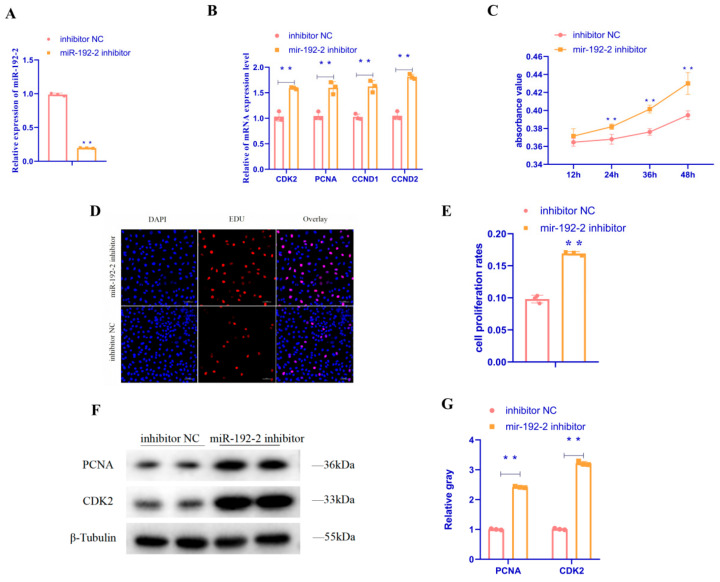
*miR-192-2* inhibitor regulates the proliferation of goose follicle granulosa cells. (**A**) The expression of *miR-192-2* in granulosa cells was detected by qRT-PCR after *miR-192-2* inhibitor or negative control (inhibitor NC) was transfected into granulosa cells. (**B**) mRNA expression of *CDK2*, *PCNA*, *CCND1*, and *CCND2* in granulosa cells transfected with *miR-192-2* inhibitor or inhibitor negative control. (**C**) Cell growth curves as measured using the CCK-8 assay following transfection with a *miR-192-2* inhibitor or inhibitor negative control in granulosa cells. (**D**,**E**) The proportion of cells in a proliferative state was determined after transfection of granulosa cells with the *miR-192-2* inhibitor or inhibitor negative control using EdU (Scale bar = 50 μm). (**F**,**G**) Abundance of proteins of *CDK2* and *PCNA* in granulosa cells transfected with *miR-192-2* inhibitor or inhibitor negative control as determined by use of Western blot analysis. Data are expressed as mean ± SEM (*n* = 3). ** *p* < 0.01.

To investigate the role of *miR-192-2* in the proliferation of primary GC in goose follicles, we transfected the *miR-192-2* mimic and *miR-192-2* inhibitor into GC, respectively, and performed qRT-PCR assays to detect the relative expression of the mRNA of *PCNA*, *CDK2*, *CCND1*, and *CCND2* in GC, CCK-8, and EdU assays to detect the proliferation status of GC, and a WB assay to detect the relative protein expression of the proliferation-related gene in GC. The expression of *CDK2*, *PCNA*, *CCND1*, and *CCND2* mRNA markedly reduced in response to the overexpression of *miR-192-2* (Figure 1A,B) (*p* < 0.01). The CCK-8 assay revealed a significant decrease in the OD value of cells at 12, 24, 36, and 48 h following the expression of *miR-192-2* (Figure 1C) (*p* < 0.01). Furthermore, the EdU assay revealed a substantial decrease in the number of positive goose follicle GC following *miR-192-2* overexpression (Figure 1D,E) (*p* < 0.01). Additionally, the expression of *CDK2 and PCNA* proteins markedly reduced after *miR-192-2* overexpression (Figure 1F,G) (*p* < 0.01). It was determined that the proliferative state of GC was significantly enhanced after the inhibition of *miR-192-2* expression. The expression of *CDK2*, *PCNA*, *CCND1*, and *CCND2* mRNA markedly elevated in response to the inhibition of *miR-192-2* expression (Figure 2A,B) (*p* < 0.01). The CCK-8 assay revealed a significant increase in the OD value of the cells after 24, 36, and 48 h of *miR-192-2* expression inhibition (Figure 2C) (*p* < 0.05). The EdU assay demonstrated a substantial increase in the number of positive GC following *miR-192-2* expression inhibition (Figure 2D,E) (*p* < 0.01). Furthermore, the expression of *CDK2* and *PCNA* proteins markedly elevated following *miR-192-2* inhibition (Figure 2F,G) (*p* < 0.01). In summary, these results demonstrated the inhibitory effect of *miR-192-2* on the proliferation of GC in goose follicles.

### 3.2. miR-192-2 Promotes Apoptosis in Primary Granulosa Cells of Ovarian Follicles

In this study, we also investigated the effect of *miR-192-2* on GC apoptosis after the transfection of the *miR-192-2* inhibitor and *miR-192-2* mimic in GC. Among them, we mainly used the qRT-PCR assay to detect the relative expression of mRNA of apoptosis-related genes in GC, a flow cytometry assay was used to detect the number of apoptotic GC, and a WB assay was used to detect the relative expression of the protein of apoptosis-related genes in GC.

**Figure 3:**
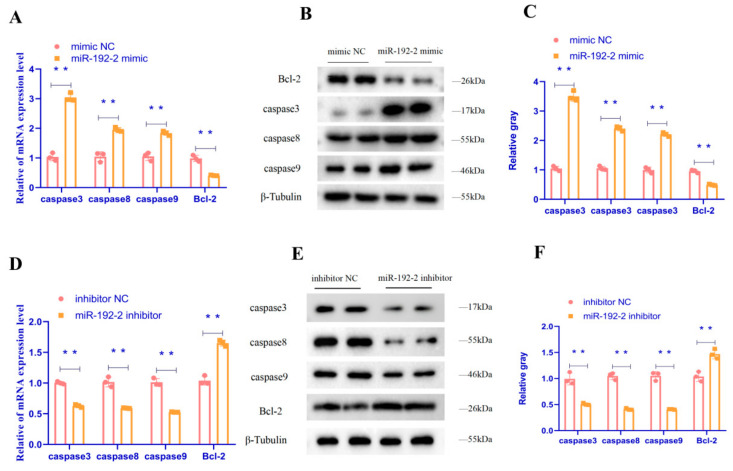

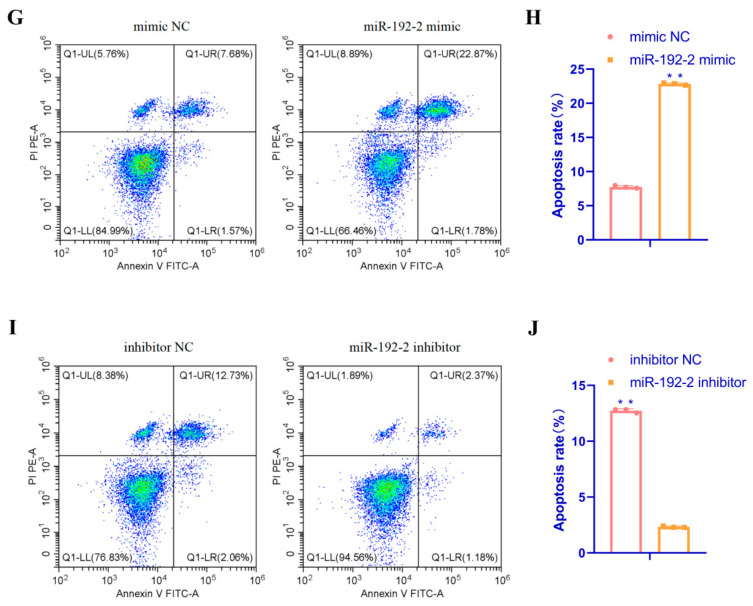
*miR-192-2* regulates apoptosis of goose follicle granulosa cells. (**A**,**D**) mRNA expression of *Caspase3*, *Caspase8*, *Caspase9*, and *Bcl-2* in GC transfected with a *miR-192-2* mimic, inhibitor, or negative control (inhibitor NC). (**B**,**C**,**E**,**F**) Abundance of proteins of *Caspase3*, *Caspase8*, *Caspase9*, and *Bcl-2* in granulosa cells transfected with *miR-192-2* mimic, inhibitor, or inhibitor negative control as determined by use of Western blot analysis. (**G**–**J**) Scatter gram and rate of apoptosis in granulosa cells transfected with *miR-192-2* inhibitor or *miR-192-2* mimics were analyzed using flow cytometry following staining with annexin V and PI. Data are expressed as mean ± SEM (*n* = 3). ** *p* < 0.01.

The present study revealed that the mRNA expression and protein expression of *Caspase3*, *Caspase8*, and *Caspase9* were significantly increased after the expression of *miR-192-2* (*p* < 0.01). Concurrently, the mRNA expression and protein expression of the anti-apoptotic gene *Bcl-2* were significantly decreased (Figure 3A–C) (*p* < 0.01). Furthermore, the results of the flow cytometry assay demonstrated that the number of apoptotic cells was significantly increased after the expression of *miR-192-2* (Figure 3G,H) (*p* < 0.05). Conversely, the apoptotic tendency of GC was significantly attenuated after the inhibition of *miR-192-2* expression. The mRNA and protein expression of *Caspase3*, *Caspase8*, and *Caspase9* were markedly reduced upon the inhibition of *miR-192-2* expression (*p* < 0.01). Conversely, the mRNA and protein expression of the anti-apoptotic gene *Bcl-2* were both considerably increased (*p* < 0. 05) (Figure 3D–F) (*p* < 0.01); the results of the flow cytometry assay showed that the number of apoptotic cells was significantly reduced after the inhibition of *miR-192-2* expression (Figure 3I,J) (*p* < 0.01). Taken together, these results demonstrated *miR-192-2* can promote apoptosis in goose follicular GC.

### 3.3. miR192-2 Targets IGFBP2 Gene

**Figure 4:**
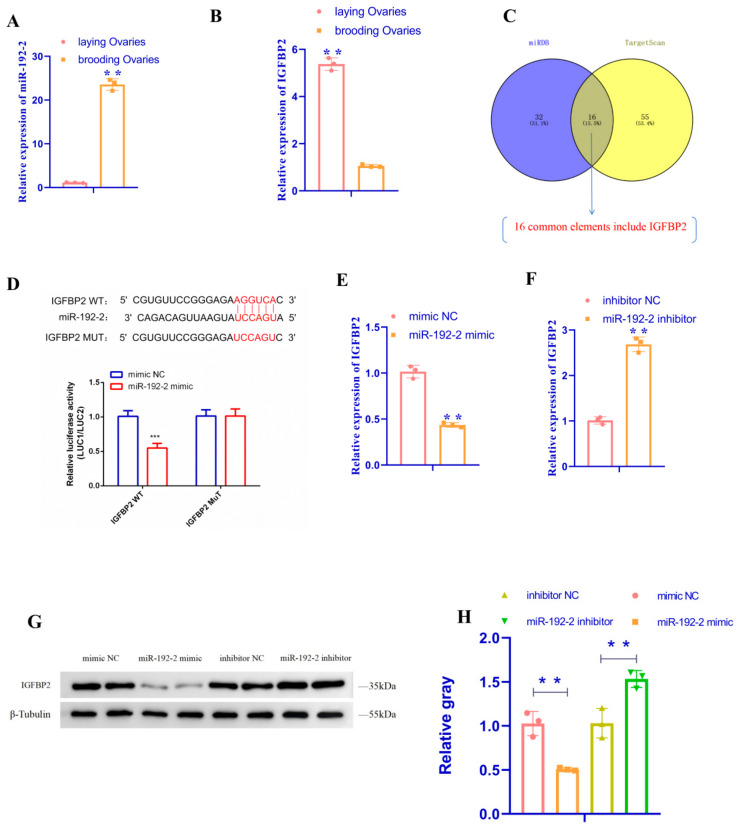
*IGFBP2* is a target gene of *miR-192-2*. (**A**,**B**) Expression of *miR-192-2* and *IGFBP2* in the ovaries of geese at the laying period and brooding period. (**C**) Predicted target genes of *miR-192-2* using TargetScan and miRDB. (**D**) *IGFBP2*-3′-UTR wild, mutant dual luciferase vectors and *miR-192-2* mimic, *miR-192-2* negative control, were co-transfected into 193T cells, and relative luciferase activity was determined after 48 h. (**E**,**F**) *IGFBP2* mRNA expression after transfection of *miR-192-2* mimic, inhibitor, or negative control (inhibitor NC) into granulosa cells. (**G**,**H**) Protein expression of *IGFBP2* after transfection of *miR-192-2* mimic, inhibitor or inhibitor NC into granulosa cells. Data are expressed as mean ± SEM (*n* = 3). ** *p* < 0.01; *** *p* < 0.001.

Insulin-like growth factor-binding protein II (*IGFBP2*) is a member of the insulin-like growth factor-binding proteins family and plays a key role in cell growth, differentiation, apoptosis, and metabolic regulation. Our previous high-throughput sequencing study of ovaries from laying and brooding geese revealed that *miR-192-2* was differentially expressed in the two types of ovaries and subsequently predicted the target genes of *miR-192-2*, and we had published one paper on the subject [26]. We then searched for all the target genes of *miR-192-2* using Miranda and TargetScan. Combining the predictions of the two bioinformatics websites to compare and evaluate target genes with which they may have a close target relationship, there were 16 common target genes (*ARFGEF1*, *BHLHE22*, *CCNT2*, *DYRK3*, *EREG*, *FRMD4B*, *IGFBP2*, *LPAR4*, *MSN*, *NIPAL1*, *PDP1*, *PKP4*, *RAB2A*, *RB1*, *WDR44*, and *ZEB2*) in the results predicted by the two bioinformatics websites, including *IGFBP2* (Figure 4C). Among all of the candidate genes, *IGFBP2* was involved in animal ovarian function and had a highly conserved *miR-192-2* binding site in its 3′-UTR. Therefore, we performed qRT-PCR assays for *miR-192-2* and *IGFBP2* in laying and brooding follicles and found that the expression of *miR-192-2* in laying follicles was significantly lower than that in brooding follicles, whereas the expression pattern of *IGFBP2* was the opposite to that of *miR-192-2* (Figure 4A,B) (*p* < 0.01). In conclusion, *IGFBP2* may be a target gene of goose *miR-192-2*. As indicated by the website-predicted *miR-192-2* binding site with *IGFBP2*, the site will be designed using a mutation sequence. The results of the dual luciferase assay demonstrated that the binding of *miR-192-2* with the wild type of *IGFBP2* resulted in a decrease in dual luciferase activity. However, the luciferase activity of the two *IGFBP2*-3′UTR-MUT co-transfected groups showed no significant change, suggesting that this site is the binding site (Figure 4D). Taken together, the results of this study suggest a targeting relationship between *miR-192-2* and *IGFBP2*. However, the expressions of *IGFBP2* mRNA and protein in GC were also analyzed after transfection with *miR-192-2* inhibitor and *miR-192-2* mimic. The results demonstrated that both the mRNA and protein expressions of *IGFBP2* were significantly elevated after the inhibition of *miR-192-2* expression (*p* < 0.01), while both the mRNA and protein expressions of *IGFBP2* were significantly reduced after the overexpression of *miR-192-2* (Figure 4E–H) (*p* < 0.01). Taken together, these results suggest a target relationship between *miR-192-2* and *IGFBP2*.

### 3.4. IGFBP2 Promotes the Proliferation of Primary GC in Ovarian Follicles

**Figure 5:**
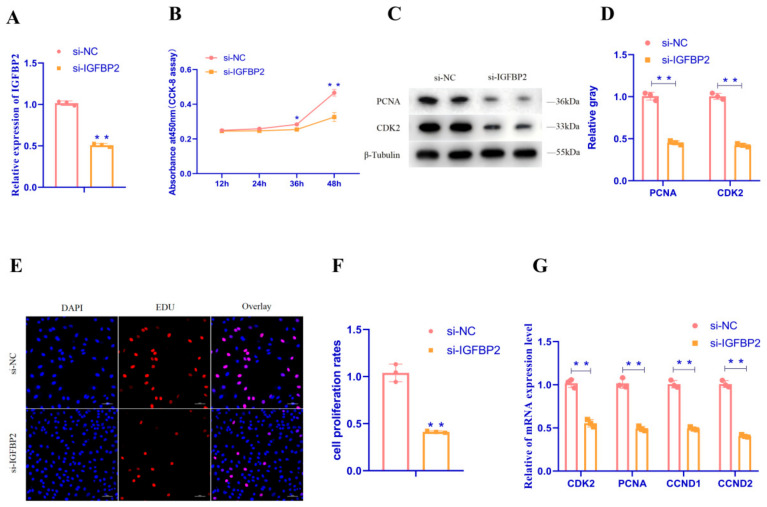
*IGFBP2* promotes granulosa cell proliferation in goose follicles. (**A**) The mRNA expression of *IGFBP2* was detected after transfection of si-*IGFBP2* or negative control (si-NC) into granulosa cells. (**B**) Cell growth curves were determined using CCK-8 after transfection of si-*IGFBP2* or si-negative control into granulosa cells. (**C**,**D**) Protein expression of *CDK2* and *PCNA* was detected after transfection of si-*IGFBP2* or si-negative control into granulosa cells. (**E**,**F**) The proportion of cells in a proliferative state was determined after transfection of si-*IGFBP2* or si-negative control into granulosa cells using EdU (Scale bar = 50 μm). (**G**) The mRNA expression of *CDK2*, *PCNA*, *CCND1*, and *CCND2* was detected after transfecting granulosa cells with si-*IGFBP2* or si-negative control. Data are expressed as mean ± SEM (*n* = 3). * *p* < 0.05, ** *p* < 0.01.

To investigate the role of *IGFBP2* in GC proliferation, we performed a qRT-PCR assay to detect the relative mRNA expression of proliferation-related genes in GC and CCK-8, an EdU assay to detect the proliferation status of GC, and a WB assay to detect the relative protein expression of proliferation-related genes in GC after the downregulation of *IGFBP2* expression in goose follicle primary GC. As demonstrated in Figure 5, the mRNA expression of *CDK2*, *PCNA*, *CCND1*, and *CCND2* (Figure 5A,G) and the protein expression of *CDK2* and *PCNA* were markedly reduced (Figure 5C,D) following the downregulation of *IGFBP2* expression in GC (*p* < 0. 01); the CCK-8 assay results demonstrated that the OD value of GC after the downregulation of *IGFBP2* expression was significantly reduced (Figure 5B) (*p* < 0.05); the EdU assay results showed that the number of positive GC was significantly reduced after the downregulation of *IGFBP2* expression (Figure 5E,F) (*p* < 0.01). These findings collectively indicated that *IGFBP2* exerted a promoting effect on the proliferation of goose follicular GC.

### 3.5. IGFBP2 Inhibits Apoptosis in Goose Follicular GC

**Figure 6:**
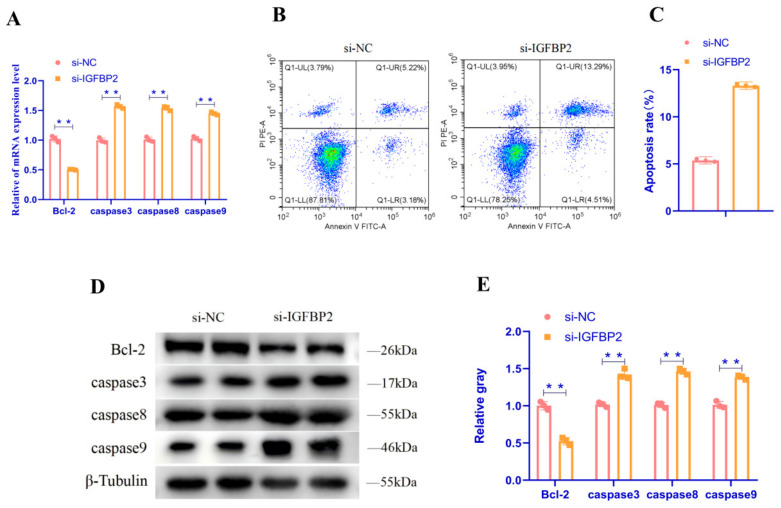
*IGFBP2* inhibits apoptosis in goose follicular granulosa cells. (**A**) The mRNA expressions of *Bcl-2*, *Caspase3*, *Caspase8*, and *Caspase9* were determined after the transfection of the interfering vector and small interfering RNA for the synthesis of *IGFBP2* (si-*IGFBP2*) or negative control (si-NC) into GC. (**B**,**C**) Scatter gram and rate of apoptosis in granulosa cells transfected with si-*IGFBP2* or si-negative control as analyzed using flow cytometry following staining with annexin V and PI. (**D**,**E**) Abundance of proteins of *Caspase3*, *Caspase8*, *Caspase9*, and *Bcl-2* in granulosa cells transfected with si-*IGFBP2* or si-negative control as determined by the use of Western blot analysis. Data are presented as mean ± SEM (*n* = 3). ** *p* < 0.01.

To explore the role of *IGFBP2* in GC apoptosis, we performed a qRT-PCR assay to detect the relative expression of mRNA of apoptosis-related genes in GC, a flow cytometry assay to detect the number of apoptotic GC, and a WB assay to detect the relative expression of proteins of apoptosis-related genes in GC after the downregulation of *IGFBP2* expression in goose follicle GC. As demonstrated in Figure 6, the downregulation of *IGFBP2* in GC resulted in a substantial increase in the mRNA and protein expression of *Caspase3*, *Caspase8*, and *Caspase9* (*p* < 0.05). Conversely, the mRNA and protein expressions of the anti-apoptotic gene *Bcl-2* were markedly reduced (Figure 5A,D,E) (*p* < 0.05). The results of the flow cytometry assay revealed a substantial increase in the number of apoptotic GC following the downregulation of *IGFBP2* expression in GC (Figure 5B,C) (*p* < 0.05). These results collectively demonstrated the inhibitory effect of *IGFBP2* on apoptosis in goose follicle GC.

## 4. Discussion

Growing evidence suggests that miRNAs are closely linked to the connection between the ovaries and GC [27]. GC are important somatic cells in the follicle [2], supporting follicular development by secreting hormones, but an imbalance in the internal homeostasis of the follicle leads to GC apoptosis, resulting in follicular atresia [28]. In avian species, follicular atresia is the main cause of reduced reproductive performance in laying birds. Therefore, the proliferation and apoptosis of GC are closely related to the laying performance of birds. It has been shown that the *miR-192* family has emerged as a key miRNA in various cancers [13]. However, there is relatively little research on its expression in bird ovarian tissue. Our previous high-throughput sequencing study on the ovaries of laying and brooding geese revealed that *miR-192-2* was differentially expressed in the two types of ovaries, suggesting that *miR-192-2* may be involved in the growth and development of goose follicles, as well as in the proliferation and apoptosis of follicular cells.

The proliferation and differentiation of GC affect primordial follicle initiation and growth and regulate the development of growing follicles through a receptor-mediated pathway. *CDK2*, *PCNA*, *CCND1*, and *CCND2* have been reported as marker genes for cell proliferation [29,30]. miRNAs are involved in the regulation of cell proliferation, and it was found that *LINC00477* inhibited the proliferation of ovarian GC by targeting *miR-128* [31]. miRNAs have been identified as critical regulators of cell proliferation. In a seminal study, Tu et al. demonstrated that the *miR-10* family members function as suppressors of ovarian GC proliferation in human, mouse, and rat ovaries [32]. Our experimental results showed that overexpression of *miR-192-2* inhibited goose follicle GC proliferation and promoted apoptosis. This was consistent with another study in which grape seed proanthocyanidin B2 was used to downregulate *miR-192* to inhibit the *Caspase3* apoptotic signaling pathway, thereby preventing damage to porcine follicular GC [18]. Similarly, our experimental results showed that overexpression of *miR-192-2* led to a significant reduction in cell viability at all times, a significant reduction in the number of positive goose follicle GC, a significant reduction in the mRNA expression of cell proliferation-related genes (*CDK2*, *PCNA*, *CCND1*, and *CCND2*), and a significant reduction in the protein expression of *CDK2* and *PCNA*. However, the proliferative state of GC was significantly improved after *miR-192-2* inhibition, and the mRNA expression of *CDK2*, *PCNA*, *CCND1*, and *CCND2* was significantly increased, and the protein expression of *CDK2* and *PCNA* was significantly increased. The results were consistent with those of CCK-8 and EdU, suggesting that *miR-192-2* inhibited the proliferation of primary GC in goose follicles.

It has been shown that miRNA is involved in a variety of biological processes, including cell growth, cell death, and cell migration. *miR-6881-3p* is involved in reducing ovarian reserve function by regulating granulosa cell apoptosis through targeting *SMAD4* [33]. The downregulation of *miR-221-3p* may be involved in the pathogenesis of diminished ovarian reserve by promoting apoptosis of GC and upregulating *FOXO1* expression [34]. The present study demonstrated that the expression of *miR-192-2* led to a significant increase in the mRNA and protein expression of apoptosis-related genes, including *Caspase3*, *Caspase8*, and *Caspase9*. Conversely, the mRNA and protein expression of the anti-apoptotic gene *Bcl-2* were significantly reduced. Furthermore, the results of the flow cytometry assay indicated that the number of apoptotic cells was significantly increased by the expression of *miR-192-2*. In summary, the results of the present study demonstrated that the expression of *miR-192-2* significantly increased the number of apoptotic cells. The apoptotic tendency of GC was significantly attenuated when *miR-192-2* expression was inhibited, suggesting that *miR-192-2* promoted apoptosis in primary GC of goose follicles.

To explore the key target genes of *miR-192-2* affecting the developmental process of GC, we used bioinformatics software to find and focus on *IGFBP2*. In this experiment, as shown by the qRT-PCR assay and the dual luciferase results, it was demonstrated that *IGFBP2* was a direct target gene of *miR-192-2*. In addition, we also verified the regulation of *IGFBP2* expression by *miR-192-2* at the mRNA and protein levels, and the results indicated a target relationship between *miR-192-2* and *IGFBP2*.

The regulation mechanism of *IGFBP2* expression is a complex multifactorial process [35]. It has an important role in regulating growth factor activity, participating in growth and development, and influencing lipid generation [36]. In addition, *IGFBP2* has been associated with a variety of diseases [37]. *IGFBP2* plays an important role in medulloblastoma proliferation, migration, signal transduction, and activation of transcription protein activity and epithelial-to-mesenchymal transition marker levels [38]. Effect of *IGFBP2* on proliferation, apoptosis, and P4 secretion in chicken follicular GC [39]. It has been suggested that *IGFBP* may regulate trophoblast cell proliferation through the *PI3K-Akt* signaling pathway, thereby affecting pregnancy outcome in pregnancy 2 [40]. Wang et al. showed that *miR-34b-5p* could mediate myoblast proliferation and differentiation by targeting *IGFBP2* [41]. To investigate the role of *IGFBP2* in GC proliferation and apoptosis, we examined the mRNA and protein expression of proliferation-related and apoptosis-related genes in GC after downregulating the expression of *IGFBP2* in primary GC of goose follicles. When the expression of *IGFBP2* was downregulated, the mRNA and protein expression of proliferation-related genes decreased in GC, whereas the mRNA expression and protein expression of apoptosis-related genes (*Caspase3*, *Caspase8*, and *Caspase9*) significantly increased, whereas the expression of mRNA and the protein of the anti-apoptosis gene *Bcl-2* significantly decreased. The results of the CCK-8 assay found that the OD value of GC was significantly reduced after downregulation of *IGFBP2* expression, and the results of the EdU assay showed that the number of positive GC was significantly reduced after downregulation of *IGFBP2* expression. The results of the flow cytometry assay also showed a significant increase in the number of apoptotic cells after *miR-192-2* overexpression. These results demonstrated the promotional effect of *IGFBP2* on the proliferation and inhibitory effect on apoptosis in goose follicular GC.

## 5. Conclusions

*miR-192-2* inhibited proliferation and promoted apoptosis of goose follicular GC. There was a target relationship between *miR-192-2* and *IGFBP2*. *IGFBP2* promoted the proliferation and inhibited the apoptosis in goose follicular GC.

## Figures and Tables

**Table 1:** Oligonucleotide sequences used in this study.

Name	Sequence (5′-3′)
*miR-192-2* mimic	AUGACCUAUGAAUUGACAGAC
	CUGUCAAUUCAUAGGUCAUUU
*miR-192-2* inhibitor	GUCUGUCAAUUCAUAGGUCAU
si-*IGFBP2*	UGAAGGAGCUGGCGGUUAUtt
mimic NC	UUGUACUACACAAAAGUACUG
inhibitor NC	CAGUACUUUUGUGUAGUACAA

**Table 2:** Primers used in this study.

Gene	Primer Sequence (5′-3′)	Size (bp)
*CDK2*	F: CCAGAACCTCCTCATCAAC	171
	R: CAGATGTCCACAGCAGTC	
*PCNA*	F: GAGACCTCAGCCACATTGGT	173
	R: AGTCAGCTGGACTGGCTCAT	
*CCND1*	F: CAGAAGTGCGAAGAGGAAGT	188
	R: CTGATGGAGTTGTCGGTGTA	
*CCND2*	F: CCCACTCGAAAGTGCCATCT	186
	R: TGCTGCAAGGTTCCACTTCA	
*Caspase3*	F: TGGCCCTCTTGAACTGAAAG	106
	R: TCCACTGTCTGCTTCAATACC	
*Caspase8*	F: AGCTGTAATGCAGGGGTTCT	197
	R: GGCCTCACGATCCTTCTGAC	
*Caspase9*	F: TCCCGGGCTGTTTCAACTT	270
	R: CCTCATCTTGCAGCTTGTGC	
*Bcl-2*	F: ATCGTCGCCTTCTTCGAGTT	150
	R: ATCCCATCCTCCGTTGTCCT	
*IGFBP2*	F: AGCGGCAGATGGGCAAAGT	184
	R: GGGGATGTGGAGGGAGTAGAGG	
*GAPDH*	F: TCCTCCACCTTTGATGCG	146
	R: GTGCCTGGCTCACTCCTT	
*U6*	F: GGGCCATGCTAATCTTCTCTGTA	
	R: CAGGTCCAGTTTTTTTTTTTTTT	
*miR-192-2*	F: GCGCGATGACCTATGAATTG	
	R: AGTGCAGGGTCCGAGGTATT	

## Data Availability

The raw data supporting the conclusions of this article will be made available by the authors on request.
